# Silencing Nogo-B receptor inhibits penile corpus cavernosum vascular smooth muscle cell apoptosis of rats with diabetic erectile dysfunction by down-regulating ICAM-1

**DOI:** 10.1371/journal.pone.0220715

**Published:** 2019-08-23

**Authors:** Yun Zhang, Wei Huo, Yan Wen, Hai Li

**Affiliations:** 1 Department of Urology, China-Japan Union Hospital of Jilin University, Changchun, P.R.China; 2 Department of Endocrine, China-Japan Union Hospital of Jilin University, Changchun, P.R. China; INSERM, Université de Bordeaux, FRANCE

## Abstract

Erectile dysfunction (ED) is a major sexual problem for men. Nogo-B receptor (NgBR) has been found to be involved in the regulation of vascular remodeling and angiogenesis. The present study explores the effects of NgBR in penile corpus cavernosum in rats with diabetic ED. Firstly, the ED model of Sprague Dawley rats was established. Hematoxylin-eosin staining and Masson staining were conducted to observe pathological morphology. Immunochemical assay was adopted to detect α-smooth muscle actin (α-SMA), NgBR and intercellular cell adhesion molecule-1 (ICAM-1) expression. Reverse transcription quantitative polymerase chain reaction assay and Western blot analysis were carried out for the assessment of NgBR, factors correlated to ICAM-1, including steroid receptor coactivator (SRC) and proline-rich tyrosine kinase2 (PYK2), and factors associated with apoptosis, including B-cell lymphoma-2 (Bcl-2), Bcl-2 associated protein X (Bax), caspase 3 and cleaved-caspase 3. The results found that capillaries and vascular smooth muscle cell content reduced, and NgBR and ICAM-1 were elevated in rats with diabetic ED. si-NgBR relieved ED by decreasing penile corpus cavernosum smooth muscle systolic percentage and increasing erectile time and rate, intracavernous pressure (ICP)/mean arterial pressure (MAP) and diastolic percentage, improving the pathological changes and inhibiting cavernosum cell apoptosis. si-NgBR also resulted in the down-regulation of ICAM-1 and downstream SRC and PYK2 and promoted α-SMA expression. In conclusion, si-NgBR can provide a potential therapy for diabetic ED in rats by down-regulating ICAM-1, SRC and PYK2, making it a potential therapeutic option for diabetic ED.

## Introduction

Erectile dysfunction (ED) is a common disease in male, which is characterized by the inability to achieve and maintain an erection for long enough to get satisfactory results from sexual intercourse, which can be a predictor of cardiovascular disease [1]. ED affects men in various age ranges, the risk of which is 9.1% for men within the ages of 40–49 years, 15.2% for 50–59 years, 29.4% for 60–69 years and 54.9% for men who are 70 or over [2]. The risk factors that contribute to the development of this condition include coronary artery disease, obesity, hypertension, depression and diabetes [3]. Importantly, the importance of diabetes, obesity, and metabolic syndrome in sexual dysfunction has also been clarified that overweight or obese men are more susceptible to ED, a frequent comorbidity of obesity [4]. In addition, ED also places a huge burden on human relationships from perspectives of society and sex and has a significant adverse effect on the life quality for patients [5]. Moreover, a functional meta-analysis or systematic review has revealed that ED in men with diabetes can be indicative of cardiovascular risk, highlighting the significance of diabetes in ED [6]. In the present study, diabetic ED rat models were used to come up with a novel therapeutic strategy for ED.

Neurite outgrowth inhibitor-B (Nogo-B) is a modulator for the motility and adhesion of vascular endothelial cells as a reticulon-4 isoform by binding to its receptor, Nogo-B receptor (NgBR) [7]. Nogo-B has been found to be a regulator for vascular remodeling and angiogenesis [8]. Furthermore, NgBR has been reported to interact with Nogo-B and to influence several pathophysiological procedures, including angiogenesis, cell proliferation and apoptosis [9], by which we inferred that its effects on angiogenesis may be applicable to ED; however, its involvement in ED remains unclear. NgBR is a cell surface receptor and plays a role by binding farnesylated Ras in order to aid the translocation of Ras to the plasma membrane [10]. NgBR has been demonstrated to be strongly related to estrogen receptor alpha and survivin in breast cancer [11]. Notably, Nogo-B is an important regulator of *in vitro* cell processes like cell migration and *in vivo* activities including vascular remodeling and angiogenesis, of which the endothelial one has been reported as a regulator for intercellular adhesion molecule-1 (ICAM-1) [12]. ICAM-1 is a transmembrane glycoprotein that belongs to the immunoglobulin superfamily, which is an important factor in signal transduction and cell adhesion [13]. There is a high expression in ICAM-1 in aging-related ED [14]. In addition, the expression of ICAM-1 has been observed to increase in penile cavernosal dysfunction that occurs as a result of chronic stress [15]. The ICAM-1 engagement has been shown to activate steroid receptor coactivator (SRC) and proline-rich tyrosine kinase2 (PYK2), both of which in return are recruited to the ICAM-1 engagement sites in human leukocytes and endothelial cells [16]. According to the biological characteristics and interaction of NgBR and ICAM-1, the present study aims to determine whether NgBR and ICAM-1 can interact with each other involving SRC and PYK2 as a way to regulate penile corpus cavernosum smooth muscle of rats with diabetic ED, in order to find a novel therapeutic approach for ED patients.

## Materials and methods

### 2.1. Ethics statement

The protocol was approved by the Institutional Animal Care and Use Committee of China-Japan Union Hospital of Jilin University. Animals would be well treated as possible as we could to relieve the pain of animals.

### 2.2. Model establishment

A total of 170 healthy Sprague Dawley (SD) rats (6 weeks old; 250–300 g; Shanghai SLAC Laboratory Animal Co., Ltd., Shanghai, China) of specific pathogen free level were collected and acclimated for 1 week; 150 SD rats were randomly selected to establish ED model [17]. Next, 10 mg/mL streptozotocin (STZ) was prepared by sodium citrate-citrate buffer solution bathed in ice. These rats were injected with 60 mg/kg STZ solution in their left lower abdominal cavity. Following the injection, blood glucose was measured by collecting blood from tail veins; this was conducted once every week, which was lowered to once every two weeks after injection for 4 weeks. After injection for 12 weeks, rats were injected with 100 μg/kg apomorphine (prepared by normal saline and 0.5% Vitamin C injection) at the cervical skin. Then, the occurrence and time of erectile were immediately observed for 30 min and recorded. Rats without hyperemia of the penis or appearance of scapus penis and with blood glucose value > 16.7 mol/L were regarded as ED rats. Following the successful establishment of the rat models, 20 rats were randomly selected as the ED group. Another 20 rats were selected as the sham group and received an injection with 0.1 M sodium citrate solution (pH = 4.5) in the abdominal cavity.

### 2.3. Rat grouping and treatment

Eighty successfully established SD rat models were selected for the following treatment. Penises of rats in the si-negative control (si-NC) group were injected with 3 μg/10 μL siRNA of non-relevant sequence (5’-TTCTXXGAACGTGTCATGT-3’) once per day; penises of rats in the si-NgBR group were injected with 3 μg/10 μL NgBR siRNA (5’-AAGGAAATACATAGACCTACA-3’) once per day [18–20]; penises of rats in the NgBR + anti-NC group were injected with the mixture of NgBR overexpression plasmid [21, 22] and normal saline once per day; penises of rats in the NgBR + anti-ICAM-1 group were injected with the mixture of NgBR overexpression plasmid and ICAM-1 antibody (100 μg, Sigma-Aldrich Chemical Company, St Louis, MO, USA). Each group had 20 rats. The penis erection rate was calculated 2 weeks later using the following formula: (the number of rats with penis erection/total number of rats in the group) × 100%.

### 2.4. Detection of intracavernous pressure (ICP)/mean arterial pressure (MAP)

Rat cervical skin was incised and the carotid artery was intubated. Next, a median incision was made on the lower abdomen to expose pelvic cavity ganglion and cavernous nerves of penis, which was later stimulated by bipolar hook-like electrodes (stimulus parameter: 5.0 mV; frequency: 20 Hz; amplitude: 5 ms; duration: 1 min). At the same time, a 23 G syringe needle containing 250 U/mL heparin was inserted into crus of penis. Two pathways were connected to pressure transducer to measure MAP and ICP values respectively and the ICP/MAP value was calculated to evaluate erectile function [23].

### 2.5. Detection of penile corpus cavernosum smooth muscle systolic and diastolic function

Penis tissues were dissected from anesthetized rats, some of which were preserved for further experiments, while the remainder was placed in 4°C Krebs solution to remove urethral cavernosum, penile cartilage, nerve and blood vessels to expose penile corpus cavernosum. The 6 mm × 2 mm × 2 mm muscle strip was prepared, one end of which was fixed in thermostatic bath and the other end was connected to ton transducer. The smooth muscle tension F2 was recorded following the equilibration of the muscle strips. The smooth muscle tension F1 was recorded after the addition of polyethylene (PE) (50 μmol/L). Next, (F1—F2)/F1 was accounted as the systolic percentage. Then PE effect was removed by rinse with Krebs solution. The smooth muscle tension F3 was recorded after the addition of Ach (100 μmol/L). Finally, (F1—F3)/F1 was accounted as the diastolic percentage.

### 2.6. Hematoxylin-eosin (HE) staining

Penis tissues were fixed by 4% paraformaldehyde, rinsed with phosphate buffer saline (PBS) and dehydrated with ethanol of gradient concentrations. The tissues were cleared with xylene, waxed, embedded with paraffin and sliced into sections (about 4 μm thick) by. These sections were dewaxed with ethanol and prepared into tissue sections for HE staining. Subsequently, the sections were stained with hematoxylin for 5 min, rinsed, stained with eosin for 2 min, dehydrated with ethanol, cleared with xylene and sealed by neutral gum. The penis tissue morphology was observed under an optical microscope and the cavernous sinus capillaries containing erythrocyte were detected, with twenty randomly selected fields. Capillary density (a capillary/mm^2^) was calculated. Some of the sections were then preserved at -20°C.

### 2.7. Masson staining

Masson staining was performed to evaluate the fibrosis of corpus cavernosum of penis. The sections were dewaxed and fixed in Bouin solution overnight, followed by Weigert iron hematoxylin staining for 5–10 min, 1% hydrochloric acid ethanol differentiation, Ponceau S acid fuchsin staining for 5–10 min and phosphomolybdic acid for 5 min. The sections were counterstained with aniline blue solution for 5 min, treated with 1% glacial acetic acid for 1 min and dehydrated with 95% ethanol several times, followed by further dehydration with anhydrous ethanol. Following xylene clearing and neutral gum sealing, collagenous fiber was observed to present in blue, elastic fiber in brown, muscle fiber, cellulose and erythrocyte in orange-red and cell nucleus in dark blue.

### 2.8. Immunohistochemistry

The 3% H_2_O_2_ was added to block endogenous enzyme. The sections were boiled in antigen retrieval buffer. After being cooled for 5 min, the heating-cooling operation was repeated twice. Then, the sections were sealed with 5% bull serum albumin (BSA) at room temperature for 20 min. After the removal of the surplus liquid, the sections were incubated at 4°C overnight with primary antibodies to rabbit anti-rat α-smooth muscle actin (α-SMA) (ab5831, 1: 3000), NgBR (ab168351, 1 : 1000), and ICAM-1 (ab171123, 1 : 1000) (all from Abcam Inc., Cambridge, MA, USA). Afterwards, the biotinylation goat anti-rabbit antibody to Immunoglobulin G (IgG) (ab172730, Abcam Inc., Cambridge, MA, USA) was added for further incubation at 37°C for 40 min. After 3 PBS rinses, streptomycin avidin-peroxidase solution (ZSGB-Bio, Beijing, China) was added and incubated with the sections at 37°C for 20 min, followed by additional 3 washes with PBS. Then, diaminobenzidine (DAB) (ZSGB-Bio, Beijing, China) was introduced for coloration. Three sections were obtained from each specimen and three fields were randomly selected from each section. Image-pro plus software (Media Cybernetics, Rockville, MD, USA) was used for quantitative analysis of the image. Expression level was measured and expressed as integral optical density (IOD) of positive staining.

### 2.9. Reverse transcription quantitative polymerase chain reaction (RT-qPCR)

Liquid nitrogen was used to cryopreserve penis tissues. Trizol assay was used to extract total RNAs in penis tissue from SD rats and total RNAs were preserved at -80°C. PrimeSeript @ RT reagent Kit (Perfect Real Time) (Takara Biotechnology Ltd., Dalian, Liaoning, China) was applied for reverse transcription of total RNAs to cDNAs, which were then stored at -20°C. Glyceraldehyde-3-phosphate dehydrogenase (GAPDH) was selected as an internal reference. RT-qPCR assay was carried out using an ABI7500 qPCR instrument (ABI Company, Oyster Bay, NY). The primers were NgBR: 5’—TGCCAGTTAGTAGCCCAGAAGCAA- 3’ (forward) and 5’—TGATGTGCCAGGGAAGAAAGCCTA- 3’ (reverse); ICAM-1: 5’—AGGTATCCATCCATCCCACA-3’ (forward) and 5’—GCCACAGTTCTCAAAGCACA—3’ (reverse); steroid receptor coactivator (SRC): 5’—CTGAGCAGATGAATGATCCA—3’ (forward) and 5’—GGACGTCAGCAAACACCTGA—3’ (reverse); PYK2: 5’—AGA TTC CCG ACG AAA CCC—3’ (forward) and 5’—CAC GGC GAA CAT CCA GAC—3’ (reverse); GAPDH: 5’—TGGTGAAGGTCGGTGTGAAC—3’ (forward) and 5’—GGTGGTGAAGACGCCAGTAG—3’ (reverse). The relative expression level was analyzed using the 2^-ΔCt^ formula.

### 2.10. Western blot analysis

Liquid nitrogen was used to cryopreserve the penis tissues and protein lysis buffer (Shanghai Genechem Co., Ltd., Shanghai, China) was used to extract total proteins. Bradford assay (Shanghai Genechem Co., Ltd., Shanghai, China) was performed for protein quantification. A total of 50 μg total proteins were separated using 12% sodium dodecyl sulfate polyacrylamide gel electrophoresis and then transferred to a polyvinylidene fluoride (PVDF) membrane (Millipore, MA, USA). After being blocked with 5% skimmed milk powder at 37°C for 1 h in a shaker, the membrane was respectively incubated with monoclonal antibodies to NgBR (ab47080), ICAM-1 (ab171123), SRC (ab47405), PYK2 (ab32571), α-SMA (ab5694), B-cell lymphoma-2 (Bcl-2) (ab32124), Bcl-2 associated protein X (Bax) (ab32503), caspase 3 (ab13585), cleaved-caspase 3 (ab2302), and GAPDH (ab8245) (all from Abcam Inc., Cambridge, MA, USA) at 4°C overnight. After being rinsed with phosphate buffered saline with Tween-20 (PBST) 3 times (5 min for each time), horseradish peroxidase labeled secondary antibody (1 : 4000; Santa Cruz Biotechnology, Inc., Santa Cruz, CA, USA) was added and incubation was carried out with the membrane at room temperature for 1 h, followed by development with enhanced chemluminesence. The relative protein expression was accounted as the ratio of gray value of target band to that of internal reference band (GAPDH).

### 2.11. Statistical analysis

All data were processed with the use of SPSS 21.0 statistical software (IBM Corp. Armonk, NY, USA). Enumeration data were expressed by rate or percentage. Chi-square test was used to compare data among multiple groups. Measurement data were expressed by mean ± standard deviation. Student *t-*test was used to compare data between two groups. One-way analysis of variance (ANOVA) was used for comparisons among multiple groups. Kolmogorov-Smirnov assay was used to assess data normality. Data obeying normal distribution were analyzed by one-way ANOVA for comparisons among multiple groups, followed by Tukey's post hoc test. Data in skewed distribution were analyzed by non-parametric Kruskal-Wallis, followed by Dunn's multiple comparison. *p* value less than 0.05 was considered to be of statistical significance.

## Results

### 3.1. NgBR and ICAM-1 are highly expressed in the diabetic ED rat model

**Fig 1:**
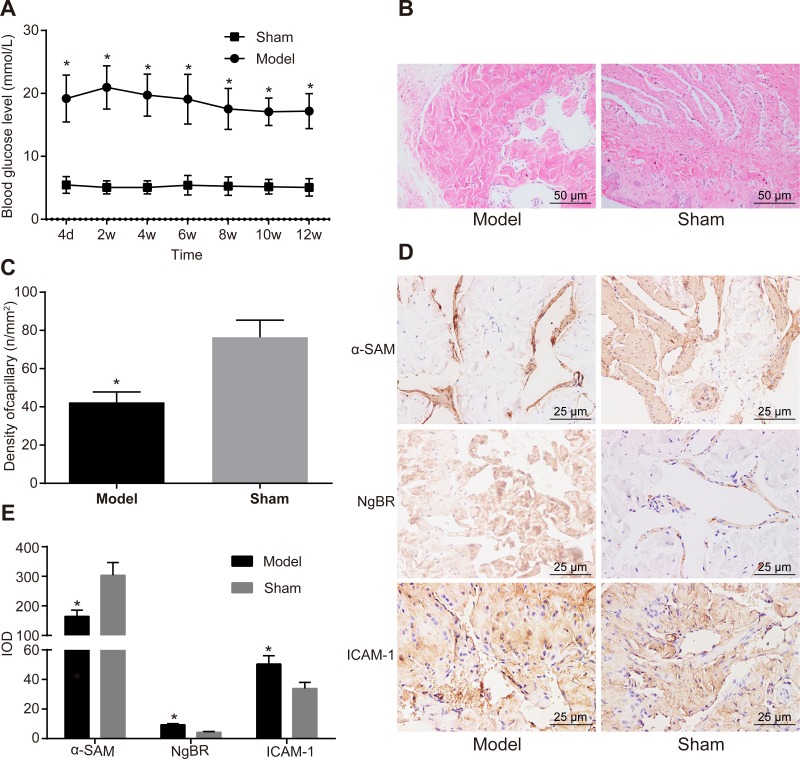
The result of immumohistochemical assay showed that NgBR and ICAM-1 expression was up-regulated in penile corpus cavernosum tissues of rats with diabetic ED. A, rats in the ED group had a higher blood glucose level than the sham group. B, HE staining for penis tissues showed that rats in the ED group had fewer capillaries than the sham group (× 200, scale bar = 50 μm). C, rats in the ED group had a lower blood vessel density of penis tissues than the sham group and statistical values were analyzed by Student *t-*test between groups. D, α-SMA was down-regulated and NgBR and ICAM-1 were overexpressed in the ED group detected by immumohistochemical assay of rat penis tissues (× 400, scale bar = 25 μm). E, the histogram showed that α-SMA was down-regulated and NgBR and ICAM-1 were overexpressed in rat penis tissues in the ED group and statistical values were analyzed by Student *t*-test between groups. * *p* < 0.05 *vs*. the sham group, n = 20.

SD rats were injected with STZ solution to establish an ED rat model. Four days later, SD rats presented with polydipsia, diuresis, emaciation, dried fur and a higher blood glucose level compared to the rats in the sham group (Fig 1A). One rat died after being administered with STZ solution at the 7th week and the 8th week, respectively. After injections for 12 weeks, 140 ED rats were successfully established, suggesting a success rate of 93.33% for model establishment. Penile corpus cavernosum tissues were stained by HE, by which cavernous sinus capillaries could be observed to contain erythrocytes and inter-weaved net-style. Penile corpus cavernosum tissues in the sham group were composed of a large number of capillaries while those in the ED group had fewer capillaries, suggesting a significant difference between the two groups (*p* < 0.05) (Fig 1B and 1C). Then, α-SMA immunohistochemical staining was used to evaluate VSMC content in cavernosum tissues. Rats in the ED group presented with evidently lowered VSMC content in cavernosum compared to the sham group (*p* < 0.05). The results from immumohistochemical assay revealed that NgBR and ICAM-1 expression in penile corpus cavernosum tissues in rats of the ED group was significantly higher than those in the sham group (*p* < 0.05) (Fig 1D and 1E). The above results suggested that the ED rat model was successfully established and NgBR and ICAM-1 were overexpressed in penile corpus cavernosum tissues of rats with diabetic ED.

### 3.2. NgBR siRNA plasmid decreases NgBR expression while NgBR overexpression plasmid increases NgBR expression

**Fig 2:**
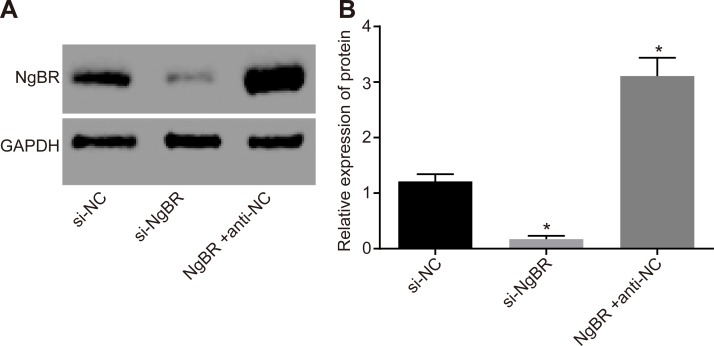
The results of Western blot analysis showed that NgBR overexpression plasmid increased NgBR expression. AB, NgBR protein level by Western blot analysis showed that the si-NgBR group had a significant decrease of NgBR expression but the NgBR + anti-NC group had an obvious increase than that in the si-NC group. The statistical values were analyzed by one-way ANOVA among multiple groups, followed by Tukey’s post hoc test. * *p* < 0.05 *vs*. the si-NC group.

Western blot analysis was used to detect transfection efficiency of NgBR siRNA and NgBR overexpression plasmid. The results showed that compared with the si-NC group, NgBR expression significantly decreased in the si-NgBR group but significantly increased in the NgBR + anti-NC group (*p* < 0.05). The results indicated that NgBR siRNA plasmid specifically silenced NgBR, and NgBR overexpression plasmid could stabilize NgBR protein level (Fig 2).

### 3.3. si-NgBR and down-regulation of ICAM-1 improve penis erectile function

Penile corpus cavernosum smooth muscle systolic and diastolic functions were assessed to observe penis erectile function of rats in all groups. Following treatment for 2 weeks, the detection results revealed that compared with the si-NC group, blood glucose level and systolic percentage significantly decreased, while erectile times and rates, ICP/MAP and diastolic percentage increased in the si-NgBR group (*p* < 0.05). The aforementioned results showed that si-NgBR could improve blood glucose and penis erectile function for rats with diabetic ED. In comparison to the NgBR+anti-NC group, blood glucose level and systolic percentage significantly decreased, while erectile times and rates, ICP/MAP and diastolic percentage were obviously elevated in the NgBR + anti-ICAM-1 group (*p* < 0.05) (Table 1). These results elucidated that ICAM-1 participated in the regulating process of ED in diabetic rats.

**Table 1:** Erectile function parameters showed that si-NgBR and ICAM-1 antibody treatments decreased blood glucose and ameliorated ED.

Groups	si-NC	si-NgBR	NgBR + anti-NC	NgBR + anti-ICAM-1
Blood glucose level (mol/L)	18.21 ± 3.42	11.91 ± 2.13*	21.21±2.33	11.54 ± 1.26^#^
Average erectile time	0.20 ± 0.41	1.85 ± 1.04*	0.25 ± 0.44	1.95 ± 0.94^#^
Number of rats with erectile	4	16*	5	17^#^
Erectile rate (%)	20	80*	25	85^#^
ICP/MAP	26.71 ± 7.56	42.31 ± 6.79*	24.39 ± 6.76	41.75 ± 9.49^#^
Systolic percentage (%)	23.75 ± 7.12	15.29 ± 6.73*	24.13 ± 9.32	17.34 ± 8.56^#^
Diastolic percentage (%)	22.13 ± 6.34	40.51 ± 7.21*	21.01 ± 7.65	37.94 ± 7.32^#^

Notes: ICP, intracavernous pressure; MAP, mean arterial pressure; blood glucose level and average erectile time were analyzed by Student *t*-test between groups; Number of rats with erectile and erectile rate were analyzed by chi-square test between groups

*, *p* < 0.05, compared with the si-NC group

#, *p* < 0.05, compared with the NgBR+ anti -NC group, n = 20.

### 3.4. si-NgBR and down-regulation of ICAM-1 attenuate pathological changes of corpus cavernosum in rats with diabetic ED

**Fig 3:**
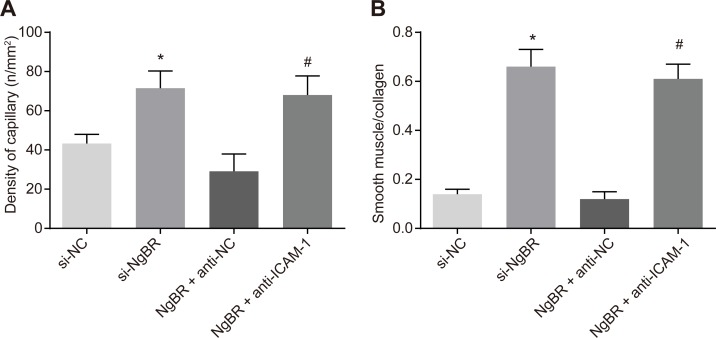
The results of HE staining, α-SMA immunochemical staining and Masson staining showed that si-NgBR could improve pathological changes in penile corpus cavernosum tissues of rats with diabetic ED and regulated by ICAM-1. A, statistical histogram showed that capillary density in penile corpus cavernosum tissues of rats treated with si-NgBR, down-regulation of ICAM-1 elevated capillary density and statistical values were analyzed by Student *t*-test between two groups. B, the ratio of smooth muscle cell to collagen in penile corpus cavernosum tissues of rats was promoted by si-NgBR and down-regulation of ICAM-1 and statistical values were analyzed by one-way ANOVA among multiple groups. * *p* < 0.05 *vs*. the si-NC group; # *p* < 0.05 *vs*. the NgBR + anti-NC group, n = 20.

HE staining was applied to penile corpus cavernosum tissue sections of rats that were euthanized 2 weeks later. Blood capillaries were observed with the density measured (Fig 3A). Masson staining was adopted to assess the degree of fibrosis of penile corpus cavernosum (Fig 3B). In the si-NC group, capillary density was decreased and VSMC content and collagen deposition was obvious, indicating penile corpus cavernosum tissue fibrosis. In comparison to the si-NC group, capillary density, and the ratio of VSMC content to collagen in cavernosum tissues were significantly elevated in the si-NgBR group (all *p* < 0.05). When compared with the NgBR + anti-NC group, capillary density, and the ratio of VSMC content to collagen were significantly elevated in the NgBR + anti-ICAM-1 group (all *p* < 0.05). The results manifested that si-NgBR could improve pathological changes in penile corpus cavernosum tissues of rats with diabetic ED, the process of which was also regulated by ICAM-1.

### 3.5. si-NgBR may down-regulate expression of ICAM-1 and its downstream SRC and PYK2

**Fig 4:**
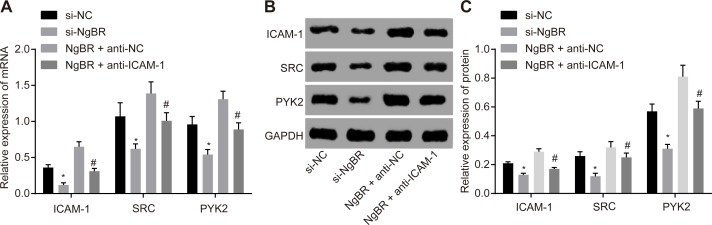
The results of RT-qPCR assay and Western analysis showed that si-NgBR down-regulated ICAM-1 and downstream SRC and PYK2. A, RT-qPCR assay showed that si-NgBR down-regulated ICAM-1 and downstream SRC and PYK2 mRNA expression. BC, Western blot analysis showed that si-NgBR down-regulated ICAM-1 and downstream SRC and PYK2 protein levels. The statistical values were analyzed by Student *t*-test. * *p* < 0.05 *vs*. the si-NC group; # *p* < 0.05 *vs*. the NgBR+ anti-NC group, n = 20.

RT-qPCR assay and Western blot analysis for penile corpus cavernosum tissue sections were applied to determine ICAM-1 and downstream SRC and PYK2 protein levels. Compared with the si-NC group, there was a significant decrease in mRNA expressions and protein levels of ICAM-1, SRC and PYK2 in the si-NgBR group (all *p* < 0.05). Compared with the NgBR + anti-NC group, mRNA expressions and protein levels of ICAM-1, SRC and PYK2 all significantly diminished in the NgBR + anti-ICAM-1 group (all *p* < 0.05) (Fig 4A–4C). These findings suggest that si-NgBR might affect rat erectile function by down-regulating ICAM-1 and its downstream SRC and PYK2.

### 3.6. si-NgBR inhibits VSMC apoptosis in rat penile corpus cavernosum by down-regulating ICAM-1

**Fig 5:**
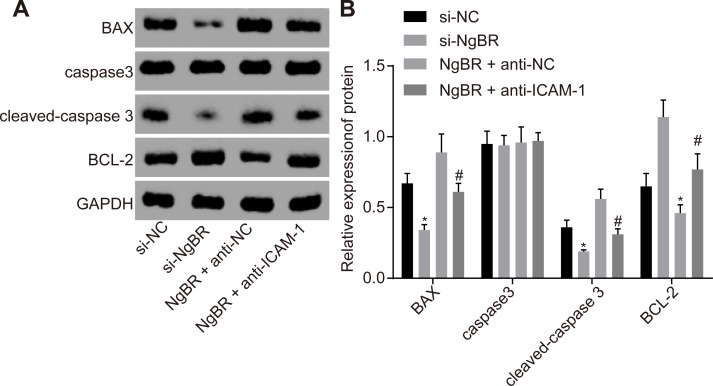
The results of Western blot analysis showed that si-NgBR and down-regulating ICAM-1 inhibited penile corpus cavernosum cell apoptosis. AB, Western blot analysis showed that si-NgBR or down-regulating ICAM-1 elevated Bcl-2 but inhibited caspase 3, cleaved-caspase 3 and Bax protein levels. The statistical values were analyzed by Student *t*-test. * *p* < 0.05 *vs*. the si-NC group; # *p* < 0.05 *vs*. the NgBR + anti-NC group, n = 20.

Western blot analysis was performed to determine protein levels of Bax, caspase 3, cleaved-caspase 3 and Bcl-2. The results from Western blot analysis revealed that compared with the si-NC group, the si-NgBR group had lower protein levels of Bax and cleaved-caspase 3, while Bcl-2 protein level was significantly higher (all *p* < 0.05). The difference regarding caspase 3 expression between the si-NC and the si-NgBR groups was considered insignificant (*p* > 0.05). These findings showed that apoptosis in the si-NgBR group was suppressed than that in the si-NC group, indicating that si-NgBR might result in the inhibition of penile corpus cavernosum cell apoptosis of rats with diabetic ED. However, compared with the NgBR + anti-NC group, the NgBR + anti-ICAM-1 group had lower protein levels of Bax and cleaved-caspase 3 but a higher Bcl-2 protein level (all *p* < 0.05) (Fig 5A and 5B). The results showed that down-regulation of ICAM-1 expression led to the inhibition of NgBR’s promoting effects on rat penile corpus cavernosum vascular smooth muscle cell apoptosis.

## Discussion

ED patients frequently present with atherosclerotic disease or other comorbid vascular disorders. [24, 25] The present study used diabetic ED rat models to evaluate the underlying molecular mechanism of ED development in order to provide a potential therapeutic option for ED. Our findings showed that NgBR and ICAM-1 were elevated in penile corpus cavernosum tissues of rats with diabetic ED, and si-NgBR or down-regulated ICAM-1 attenuated penile corpus cavernosum VSMC apoptosis of rats with diabetic ED.

One of our findings revealed an overexpression of NgBR and ICAM-1 in penile corpus cavernosum tissues of rats with diabetic ED, and silencing of NgBR decreased high blood glucose and attenuated erectile function of rats with diabetic ED. A decrease in penile vascular resistance can result in an increase in penile blood flow, the process of which is widely known as a way to achieve penile erection. [26] Cyclic guanosine monophosphate that is present in VSMCs of penis can also improve penile erection [27] It has been reported that the changes of content of cavernosal smooth muscle were related to the deterioration of ED; particularly in patients who suffer from veno-occlusive dysfunction, the erectile flow rates might be involved with the values of cavernosal smooth muscle content. [28] Nogo-B/NgBR is mainly located in the vasculature whose receptor NgBR, also known as nuclear undecaprenyl pyrophosphate synthase 1 Homolog (NUS1), has been reported to inhibit platelet derived growth factor-regulated VSMC migration. [29] NgBR is also reported to be involved in the nervous system, and blocking NgBR expression in the neuron would lead to the loss-of-Nogo branching phenotype. [30] ICAM-1 expression is upregualted by vascular endothelial growth factor in endothelial cells during inflammation through activation of nuclear factor-kB with phosphatidylinositol 39-kinase-mediated inhibition. [31] These findings were confirmed that the potential ways to treat ED may be involved in si-NgBR, and in our study, the data suggesting that capillary density, the ratio of VSMCs and collagen were all significantly elevated by silencing NgBR or down-regulating ICAM-1, may provide a potential target for therapy in ED patients.

We also found that si-NgBR inhibited penile corpus cavernosum VSMC apoptosis of rats with diabetic ED by down-regulating ICAM-1, decreasing downstream SRC and PYK2 and lowering Bax and caspase 3 expressions. In addition, si-NgBR led to decreased penile corpus cavernosum smooth muscle systolic percentage, and improved the time and rate of erection, as well as ICP/MAP and diastolic percentage. The physiological SRC proto-oncogene is a protein-tyrosine kinase that plays an important role in cell growth, division, migration, and survival signaling pathways. There exists a correlation between SRC and malignant transformation and oncogenesis as a kind of non-receptor protein-tyrosine kinase. [32] PYK2 that belongs to focal adhesion kinase family is also known as non-receptor-type proline-rich protein tyrosine kinase 2 and is mainly expressed in brain cells, hematopoietic cells and osteocytes. [33] PYK2 plays an important role in sexual function such as sperm incorporation by being recruited to the oocyte cortex, incorporating the sperm head via actin and responding to sperm binding. [34] Allingham MJ *et al*. investigated the function of tyrosine phosphorylation in the mediation of junctions during leukocyte transendothelial migration and found that ICAM-1 is involved in the mediation of SRC- and PYK2-dependent vascular endothelial cadherin tyrosine phosphorylation. [35] Moreover, a previous study has also demonstrated that Nogo-B regulates ICAM-1-mediated leukocyte transmigration and acute inflammation, which provides further evidence regarding the correlation of NgBR and ICAM-1. [12] Inflammatory cytokines such as TNF-α as well as macrophage migration activities have been demonstrated to be promoted by the overexpression of Nogo-B that participates in immune responses by modulating macrophage recruitment [36]. The up-regulation of Nogo-B is also a factor that can result in apoptosis of VSMCs [37]. In line with our results, siRNA-mediated knockdown of NgBR has been indicated to suppress survivin, an apoptosis inhibitor, in the context of breast cancer, the mechanism of which may be a potential marker for treatment modalities [9]. Decreased levels in ICAM-1 in rats with unpredictable chronic mild stress-induced ED have been suggested to be involved with the therapeutic effects of etanercept [15], which provides supporting evidence that the silencing of NgBR or down-regulation of ICAM-1 can be used to develop target treatments for ED.

In conclusion, the present study suggests that silencing NgBR or down-regulating ICAM-1 attenuated penile corpus cavernosum VSMC apoptosis of rats with diabetic ED, which could be a potential therapeutic target to alleviate erectile function. Functionally, down-regulation of ICAM-1 expression attenuated NgBR’s promoting effects on rat penile corpus cavernosum cell apoptosis. However, the present study was based on a SD rat model and the potential for translational applications for patients were not investigated. In addition, the current study mainly discussed rats with diabetic ED, which can never substitute for ED induced by hypertension, obesity and coronary artery disease. Therefore, we will continue to conduct more studies to examine the underlying mechanism in ED induced by other causes in order to provide a better therapeutic method for ED patients.

## References

[1] Compostella L, Compostella C, Truong LV, Russo N, Setzu T, Iliceto S, et al History of erectile dysfunction as a predictor of poor physical performance after an acute myocardial infarction. Eur J Prev Cardiol. 2017; 24(5):460–467. 10.1177/2047487316686434 28067536

[2] Patel JP, Lee EH, Mena-Hurtado CI, Walker CN. Evaluation and Management of Erectile Dysfunction in the Hypertensive Patient. Curr Cardiol Rep. 2017;19(9):89 10.1007/s11886-017-0889-z 28836189

[3] Patel JP, Lee EH, Mena-Hurtado CI, Walker CN. A Curr Cardiol Rep. 2017;19(9):89 -based study on prevalence and correlates of erectile dysfunction among Kinondoni District Residents, Dar Es Salaam, Tanzania, Reprod Health. 13 (2016) 140. 10.1007/s11886-017-0889-z 27899129 PMC5129661

[4] Maiorino MI, Bellastella G, Giugliano D, Esposito K. From inflammation to sexual dysfunctions: a journey through diabetes, obesity, and metabolic syndrome. J Endocrinol Invest. 2018;41(11):1249–1258. 10.1007/s40618-018-0872-6 29549630

[5] Seid A, Gerensea H, Tarko S, Zenebe Y, Mezemir R, Prevalence and determinants of erectile dysfunction among diabetic patients attending in hospitals of central and northwestern zone of Tigray, northern Ethiopia: a cross-sectional study. BMC Endocr Disord. 2017;17(1):16 10.1186/s12902-017-0167-5 28298205 PMC5353861

[6] Kouidrat Y, Pizzol D, Cosco T, Thompson T, Carnaghi M, Bertoldo A, High prevalence of erectile dysfunction in diabetes: a systematic review and meta-analysis of 145 studies. Diabet Med. 2017; 34(9):1185–1192. 10.1111/dme.13403 28722225

[7] Teng RJ, Rana U, Afolayan AJ, Zhao B, Miao QR, Konduri GG. Konduri, Nogo-B receptor modulates angiogenesis response of pulmonary artery endothelial cells through eNOS coupling. Am J Respir Cell Mol Biol. 2014 8;51(2):169–77. 10.1165/rcmb.2013-0298OC 24568601 PMC4148038

[8] Wu D, Zhao B, Qi X, Peng F, Fu H, Chi X, et al Nogo-B receptor promotes epithelial-mesenchymal transition in non-small cell lung cancer cells through the Ras/ERK/Snail1 pathway. Cancer Lett. 2018 4 1;418:135–146. 10.1016/j.canlet.2018.01.030 29331415 PMC7385903

[9] Long SL, Li YK, Xie YJ, Long ZF, Shi JF, Mo ZC. Neurite Outgrowth Inhibitor B Receptor: A Versatile Receptor with Multiple Functions and Actions. DNA Cell Biol. 2017;36(12):1142–1150. 10.1089/dna.2017.3813 29058484

[10] Jin Y, Hu W, Liu T, Rana U, Aguilera-Barrantes I, Kong A, et al Nogo-B receptor increases the resistance of estrogen receptor positive breast cancer to paclitaxel.Cancer Lett. 2018;419:233–244. 10.1016/j.canlet.2018.01.054 29373839 PMC5821135

[11] Wang B., Zhao B., North P., Kong A., Huang J., Miao Q.R., Expression of NgBR is highly associated with estrogen receptor alpha and survivin in breast cancer, PLoS One. 8 (2013) e78083 10.1371/journal.pone.0078083 24223763 PMC3817177

[12] Di Lorenzo A, Manes TD, Davalos A, Wright PL, Sessa WC. Endothelial reticulon-4B (Nogo-B) regulates ICAM-1-mediated leukocyte transmigration and acute inflammation, Blood. 2011;117(7):2284–95. 10.1182/blood-2010-04-281956 21183689 PMC3062334

[13] Usami Y, Ishida K, Sato S, Kishino M, Kiryu M, Ogawa Y, et al Toyosawa, Intercellular adhesion molecule-1 (ICAM-1) expression correlates with oral cancer progression and induces macrophage/cancer cell adhesion. 2013 8 1;133(3):568–78. 10.1002/ijc.28066 23364881

[14] Demirtaş Şahin T, Yazir Y, Utkan T, Gacar G, Furat Rençber S, Gocmez SS. TNF-alpha antagonism with etanercept enhances penile NOS expression, cavernosal reactivity, and testosterone levels in aged rats, Can J Physiol Pharmacol. 2018;96(2):200–207. 10.1139/cjpp-2017-0113 29260891

[15] Demirtaş Şahin T, Yazir Y, Utkan T, Gacar G, Halbutoğulları ZS, Gocmez SS. Depression induced by chronic stress leads to penile cavernosal dysfunction: protective effect of anti-TNF-alpha treatment. Can J Physiol Pharmacol. 2018;96(9):933–942. 10.1139/cjpp-2017-0778 30052465

[16] Allingham MJ, van Buul JD, Burridge K. ICAM-1-mediated, Src- and Pyk2-dependent vascular endothelial cadherin tyrosine phosphorylation is required for leukocyte transendothelial migration. J Immunol. 2007;179(6):4053–64. 10.4049/jimmunol.179.6.4053 17785844

[17] Chen Y, Li XX, Lin HC, Qiu XF, Gao J, Dai YT, et al The effects of long-term administration of tadalafil on STZ-induced diabetic rats with erectile dysfunction via a local antioxidative mechanism. Asian J Androl. 2012 7;14(4):616–20. 10.1038/aja.2012.22 22504870 PMC3720091

[18] Zhang Z, Zhang HY, Zhang Y, Li H. Inactivation of the Ras/MAPK/PPARgamma signaling axis alleviates diabetic mellitus-induced erectile dysfunction through suppression of corpus cavernosal endothelial cell apoptosis by inhibiting HMGCS2 expression.Endocrine. 2019;63(3):615–631. 10.1007/s12020-018-1810-2 30460485

[19] Zhang Y, Jia LP, Zhang Y, Ji W, Li H, Angiotensin II Silencing Alleviates Erectile Dysfunction Through Down-Regulating the Rhoa/Rho Kinase Signaling Pathway in Rats with Diabetes Mellitus. 2018;45(1):419–427. 10.1159/000486919 29402797

[20] Magee TR, Kovanecz I, Davila HH, Ferrini MG, Cantini L, Vernet D, et al Antisense and short hairpin RNA (shRNA) constructs targeting PIN (Protein Inhibitor of NOS) ameliorate aging-related erectile dysfunction in the rat, J Sex Med. 2007 5;4(3):633–643. 10.1111/j.1743-6109.2007.00459.x 17433082

[21] Miao RQ, Gao Y, Harrison KD, Prendergast J, Acevedo LM, Yu J, et al Identification of a receptor necessary for Nogo-B stimulated chemotaxis and morphogenesis of endothelial cells, Proc Natl Acad Sci U S A. 2006;103(29):10997–1002. 10.1073/pnas.0602427103 16835300 PMC1544163

[22] Burchardt M, Burchardt T, Anastasiadis AG, Buttyan R, de la Taille A, Shabsigh A, et al Application of angiogenic factors for therapy of erectile dysfunction: protein and DNA transfer of VEGF 165 into the rat penis, Urology. 2005;66(3):665–70. 10.1016/j.urology.2005.03.058 16140112

[23] Sun C, Lin H, Yu W, Li X, Chen Y, Qiu X, et al Neurotrophic effect of bone marrow mesenchymal stem cells for erectile dysfunction in diabetic rats. Int J Androl. 2012;35(4):601–7. 10.1111/j.1365-2605.2012.01250.x 22428616

[24] Aversa A, Greco E, Bruzziches R, Pili M, Rosano G, Spera G. Relationship between chronic tadalafil administration and improvement of endothelial function in men with erectile dysfunction: a pilot study, Int J Impot Res. 2007;19(2):200–7. 10.1038/sj.ijir.3901513 16943794

[25] Ryu JK, Jin HR, Yin GN, Kwon MH, Song KM, Choi MJ, et al Erectile dysfunction precedes other systemic vascular diseases due to incompetent cavernous endothelial cell-cell junctions, J Urol. 2013;190(2):779–89. 10.1016/j.juro.2013.02.100 23454152

[26] Yang R, Wang J, Chen Y, Sun Z, Wang R, Dai Y. Effect of caffeine on erectile function via up-regulating cavernous cyclic guanosine monophosphate in diabetic rats. J Androl. 2008;29(5):586–91. 10.2164/jandrol.107.004721 18421070

[27] Francis SH, Morris GZ, Corbin JD. Molecular mechanisms that could contribute to prolonged effectiveness of PDE5 inhibitors to improve erectile function, Int J Impot Res. 2008;20(4):333–42. 10.1038/ijir.2008.4 18418391

[28] Wespes E.Smooth muscle pathology and erectile dysfunction, Int J Impot Res. 2002;14 Suppl 1:S17–21. 11850730 10.1038/sj.ijir.3900792

[29] Teng R.J. Nogo-B Receptor (NgBR): A New Receptor that Modulates Blood Vessel Formation, Receptors & Clinical Investigation. 2014; 1:e144.

[30] Eckharter C, Junker N, Winter L, Fischer I, Fogli B, Kistner S. Schwann Cell Expressed Nogo-B Modulates Axonal Branching of Adult Sensory Neurons Through the Nogo-B Receptor NgBR. Front Cell Neurosci. 2015;9:454 10.3389/fncel.2015.00454 26635533 PMC4655273

[31] Kim I, Moon SO, Kim SH, Kim HJ, Koh YS, Koh GY. Vascular endothelial growth factor expression of intercellular adhesion molecule 1 (ICAM-1), vascular cell adhesion molecule 1 (VCAM-1), and E-selectin through nuclear factor-kappa B activation in endothelial cells. J Biol Chem. 2001;276(10):7614–20. 10.1074/jbc.M009705200 11108718

[32] Roskoski R Jr. Src protein-tyrosine kinase structure, mechanism, and small molecule inhibitors, Pharmacol Res. 2015;94:9–25. 10.1016/j.phrs.2015.01.003 25662515

[33] Suzuki C, Nakamura A, Miura N, Fukai K, Ohno N, Yahata T, et al, Non-receptor type, proline-rich protein tyrosine kinase 2 (Pyk2) is a possible therapeutic target for Kawasaki disease, Clin Immunol. 2017;179:17–24. 10.1016/j.clim.2017.01.013 28167306

[34] Wang H, Luo J, Carlton C, McGinnis LK, Kinsey WH. Sperm-oocyte contact induces outside-in signaling via PYK2 activation, Dev Biol. 2017;428(1):52–62. 10.1016/j.ydbio.2017.05.016 28527703 PMC5539980

[35] Allingham MJ, van Buul JD, Burridge K. ICAM-1-mediated, Src- and Pyk2-dependent vascular endothelial cadherin tyrosine phosphorylation is required for leukocyte transendothelial migration. J Immunol. 2007 9 15;179(6):4053–64. 10.4049/jimmunol.179.6.4053 17785844

[36] Zhu Y, Tong Q, Ye J, Ning Y, Xiong Y, Yang M, et al Nogo-B Facilitates LPS-Mediated Immune Responses by Up-Regulation of TLR4-Signaling in Macrophage RAW264.7. Cell Physiol Biochem. 2017;41(1):274–285. 10.1159/000456094 28214833

[37] Zheng H, Xue S, Lian F, Wang YY. 2011 Aug;77(2):278–81. A novel promising therapy for vein graft restenosis: overexpressed Nogo-B induces vascular smooth muscle cell apoptosis by activation of the JNK/p38 MAPK signaling pathway. Med Hypotheses. 2011 8;77(2):278–81. 10.1016/j.mehy.2011.04.035 21612877

